# Multi‐Experiment and Multi‐Locus Genome‐Wide Association Mapping for Grain Arsenic in Rice Population

**DOI:** 10.1002/pld3.70064

**Published:** 2025-05-04

**Authors:** Caijin Chen, Panthita Ruang‐areerate, Anthony J. Travis, Alex Douglas, David E. Salt, Shannon R. M. Pinson, Georgia C. Eizenga, Adam H. Price, Gareth J. Norton

**Affiliations:** ^1^ School of Biological Sciences University of Aberdeen Aberdeen UK; ^2^ Department of Plant Science University of Cambridge Cambridge UK; ^3^ National Omics Center National Science and Technology Development Agency (NSTDA) Pathum Thani Thailand; ^4^ Institute of Applied Health Sciences University of Aberdeen Aberdeen UK; ^5^ School of Biosciences University of Nottingham Sutton Bonington UK; ^6^ Dale Bumpers National Rice Research Center USDA Agricultural Research Service Stuttgart Arkansas USA

**Keywords:** candidate genes, genome‐wide association mapping, grain arsenic, quantitative trait loci, rice, Rice Diversity Panel 1

## Abstract

Rice is a globally important crop and is particularly efficient at assimilating arsenic (As). Identifying QTLs and genes associated with grain As is essential for breeding low‐As rice cultivars. In this study, data on As accumulation in grains of Rice Diversity Panel 1 in five field environments at four diverse geographic sites were reanalyzed to compare genome‐wide association (GWA) methods. Two single‐locus (EMMAX for single trait and GEMMA for multi‐experiments) and six multi‐locus (FASTmrEMMA, ISIS EM‐BLASSO, mrMLM, pKWmEB, pLARmEB, and FASTmrMLM) GWA methods were used. A total of 90 and 111 QTLs were detected using EMMAX and GEMMA, respectively. A total of 2, 11, 12, 19, 23, and 25 QTNs were identified by FASTmrEMMA, ISIS EM‐BLASSO, mrMLM, pKWmEB, pLARmEB, and FASTmrMLM, respectively. Among these, 22 QTLs/QTNs were co‐detected by single‐locus and multi‐locus GWAS methods. From these QTLs/QTNs, a total of 10 candidate genes were identified. Analysis of the haplotype variants of one candidate genes, *OsABCC1*, and one cluster of the plasma membrane intrinsic proteins *genes* revealed that a greater than 10% reduction in grain As could be achieved. The QTLs/QTNs and candidate genes identified give insight into the molecular mechanisms regulating As accumulation in rice and serve as breeding targets for developing low grain As rice cultivars.

## 1 Introduction

Rice is one of the most important crops globally, providing the calorific needs of millions of people daily (McNally et al. 2009). However, rice also accumulates a higher concentration of arsenic (As) than other staple cereals such as wheat and barley (Williams et al. 2007). Of note, due to expense, most studies measure total As concentration that includes both inorganic As, the more toxic form, and organic As, which is less toxic to humans. The World Health Organization (WHO) has established FAO/CODEX limits only for inorganic As (Codex Alimentarius 2017). Inorganic As includes both the trivalent form arsenite, As(III), which is highly soluble in water, and the more toxic pentavalent form arsenate, As(V), which is less water soluble. Rice is efficient at As accumulation as it is predominantly grown in flooded paddy soils, which leads to As(III) mobilization, and the inadvertent yet sometimes high uptake of As(III) through the highly effective silicon transport pathway found in rice (Etesami et al. 2023; F. Zhao et al. 2010). Arsenic contamination of soil due to irrigation with As‐rich groundwater or mining activities in Asia has resulted in further elevation of As levels in rice (Meharg and Rahman 2003; Williams et al. 2006; Zhu et al. 2008). While not all rice contains concerning levels of As, long‐term consumption of rice contaminated with high levels of As has been linked to multiple health problems such as cardiovascular disease risk, skin lesions, and squamous cell skin cancers (Karagas et al. 2019; Rahman et al. 2023). Both preharvest and postharvest mitigation approaches have been proposed to decrease As content in cooked rice, but leveraging the natural variation that exists within rice to develop cultivars that limit accumulation of As in rice grains may also prove effective.

Several quantitative trait loci (QTLs) for As accumulation in rice have been identified (Dong et al. 2023; Jian et al. 2008; X. Liu et al. 2019; H. Liu et al. 2021; Murugaiyan et al. 2019; Norton et al. 2014, 2019; Pinson et al. 2022; Yang et al. 2018; J. Zhang et al. 2008). For example, a QTL on Chromosome 2 for As concentration in the shoots and two QTLs on Chromosomes 6 and 8 for As concentration in the grain, identified in F1 plants from a cross between a *japonica* cultivar, CJ06, and an *indica* cultivar, TN1 (J. Zhang et al. 2008). In addition, two QTLs for As concentration in roots have been mapped on Chromosome 8, and six QTLs mapped to Chromosomes 2, 5, 6, and 9, from a backcross breeding population (Murugaiyan et al. 2019). A total of 22 QTLs have been identified for grain As based on genome‐wide association (GWA) mapping of 276 rice accessions from the Rice Diversity Panel 1 (RDP1), a collection of rice cultivars from 10 geographic regions where rice is grown (X. Liu et al. 2019) and 74 QTLs for grain arsenic identified by GWA mapping using the Bengal and Assam Aus Panel (BAAP) (Norton et al. 2019). Moreover, genes involved in the uptake and transport of As in rice have been reported, such as *Lsi1* and *Lsi2*, which are transporters belonging to the nodulin 26‐like intrinsic proteins (NIPs) subfamily of aquaporins. *OsLsi1* (*OsNIP2;1*) is a passive aquaporin channel with permeability to several substrates including silicon and As, and mutation of *Lsi1* significantly decreased As uptake (Jian et al. 2008; Mitani et al. 2008; Mitani‐Ueno et al. 2011). *OsLsi2* is an active efflux transporter for silicon and As, where mutation of *Lsi2* significantly decreased As transport to the xylem and accumulation in shoots and grains (Jian et al. 2008). Additionally, several NIPs (*OsNIP1;1*, *OsNIP2;2* (*OsLsi6*), *OsNIP3;1*, *OsNIP3;2*, and *OsNIP3;3*) have been reported to have an impact on As(III) accumulation (Jian et al. 2008; Sun et al. 2018). Members of the plasma membrane intrinsic proteins (PIP) subfamily, *OsPIP2;4*, *OsPIP2;6*, and *OsPIP2;7*, have also been implicated in As(III) tolerance and transport (Mosa et al. 2012). Phosphate transporters *OsPT1*, *OsPT4*, and *OsPT8* are also capable of As(V) uptake (Kamiya et al. 2013; Ye et al. 2017; P. Wang, Zhang, et al. 2016). After being taken up by rice roots, As(V) can be readily reduced to As(III) by *OsHAC1;1*, *OsHAC1;2*, and *OsHAC4* (Shi et al. 2016; J. Xu et al. 2017). The rice ABC transporter *OsABCC1* has been reported to sequester As(III) into the vacuoles of phloem companion cells in stem nodes, and thus reduce As accumulation in rice grain (Song et al. 2014). It is important to appreciate that post uptake compartmentalization of arsenic is critically important in determining grain arsenic content (Pinson et al. 2022).

GWA mapping identifies associations between nucleotide polymorphisms and phenotypic variance using a diverse population. Based on these genomic associations, candidate genes associated with phenotypic traits have been identified in many crops, such as maize (M. Wang et al. 2012), rice (Huang et al. 2010, 2012), and soybean (Z. Liu et al. 2020). GWA mapping for phenotypic traits in plants typically identifies associations between a single trait and single locus (univariate) based on an individual experiment (Chen, Norton, and Price, 2020, Chen et al., 2021; Norton et al. 2019), and numerous statistical models have been developed, such as the Efficient Mixed‐Model Association eXpedited (EMMAX) (Kang et al. 2010) and Genome‐Wide Efficient Mixed‐Model Association (GEMMA) (Zhou and Stephens 2014). However, a limitation of this approach is inconsistent QTL identification due to high environmental variation between experiments (e.g. temperature, humidity, and soil conditions) and the complex genetic architecture of the population, for example, rare variants in genes of large effect and/or common variation in genes of small effect (Korte and Farlow 2013). Multivariate linear mixed models (mvLMMs), which relate explanatory variables to multiple correlated outcome variables, have become increasingly important in GWA studies due to their effectiveness in accounting for relatedness among samples and population stratification and a growing appreciation of the power gains of mvLMMs over standard univariate analysis (Zhou and Stephens 2014). However, single‐locus methods may fail to capture the true genetic model of complex traits, particularly due to the significant experimental errors inherent in field studies of crop genetics (Li et al. 2022; Y.M. Zhang et al. 2019). Additionally, inflation of the Type I error rate (false positives) is commonly observed with single‐locus approaches and needs to be accounted for post model fitting (Kaler et al. 2020). Multi‐locus GWA mapping methods have been recommended to overcome these problems, including the random‐SNP‐effect mixed linear model (mrMLM) (S. Wang, Feng, et al., 2016), fast MLM (FASTmrMLM) (Tamba and Zhang 2018), fast multi‐locus random‐SNP‐effect efficient mixed model analysis (FASTmrEMMA) (Wen et al. 2018), polygenic‐background‐control–based least angle regression plus empirical Bayes (pLARmEB) (J. Zhang et al. 2017), integration of Kruskal–Wallis test with empirical Bayes (pKWmEB) (Ren et al. 2018), and iterative modified‐sure independence screening expectation–maximization‐Bayesian least absolute shrinkage and selection operator (ISIS EM‐BLASSO) (Tamba et al. 2017).

The RDP1 is a rice panel representing a broad range of rice cultivars from over 70 countries (Eizenga et al. 2014). D. Wang, Agosto‐Pérez, et al. (2018) imputed 5,231,435 SNPs for RDP1 by comparing the 700,000 SNPs (McCouch et al. 2016) with whole‐genome sequence data of the 3000 sequenced rice cultivars (W. Wang, Mauleon, et al. 2018). Previously GWA mapping was conducted on the grain As phenotype characterized on approximately 370 rice cultivars from RDP1 grown in three countries with five different field locations/years (Norton et al. 2014); however, this QTL analysis used a single GWA method and a limited number of SNPs (44K). The objective of this study was to identify the QTLs and candidate genes associated with variance for grain As concentration within the RDP1 population by different GWA mapping methods using 5.2 million SNPs.

## 2 Methods and Materials

### 2.1 Material

A total of 253 rice accessions from the RDP1 (Eizenga et al. 2014) were used in this study (Table S1), with this subset from the larger RDP1 being defined as those RDP1 accessions for which there were data from all five field experiments (seeds of these accessions are publicly available from the Genetic Stocks‐*Oryza* collection at www.ars.usda.gov/GSOR). This diverse population comprises genotypes from the five rice subpopulations: *indica*, *aus*, *aromatic*, *tropical japonica*, and *temperate japonica*. The publicly available 5.2 million–SNP database (www.ricediversity.org/data/) (D. Wang, Agosto‐Pérez, et al. 2018) of RDP1 was generated by imputing from the set of the intersection of 700k SNPs from the High Density Rice Array (HDRA; McCouch et al. 2016) and 18M SNPs from the resequencing data of the 3000 Rice Genome Project (W. Wang, Mauleon, et al. 2018), resulting in 4.8M high‐quality HDRA‐imputed SNPs (D. Wang, Agosto‐Pérez, et al. 2018). SNPs with minor allele frequency (MAF) < 0.05 were filtered out and maximum per‐SNP missing was set at 5% and a total of 3.5 million SNPs were kept after filtering.

### 2.2 Field Screen and Arsenic Analysis

The field screening for grain As was conducted in four locations: Bangladesh, China, and two US field locations (Arkansas and Texas). Soil chemistry and field management details for the production and harvest of the RDP1 grains analyzed were described in Norton et al. (2014) where single‐locus GWA mapping using 44k SNP markers was conducted. Below is a summary of the field experiments. The field site in Bangladesh was located at Faridpur in 2009; the field site in China was located at Qiyang, Hunan province, in 2009. At both sites, the plants were transplanted in a randomized complete block design with four replicates and were maintained under continuous flooding until most of the cultivars had flowered, at which time the field was drained and allowed to dry before harvest of the grains. For the field site in Arkansas, the cultivars were grown in year 2006 and 2007; the field layout in both years was a randomized complete block design with two replicates. The flood was applied when the plants were at the five‐leaf stage and drained 15–20 days after the majority of the cultivars had flowered to allow fields to dry before harvest. For the field site in Texas, three replicates of the RDP1 accessions were grown in 2009 using a randomized complete block design. The fields were flush‐irrigated until plants reached an average height of 18 cm. At this stage, a 10‐cm permanent flood was established and maintained until the seeds of all genotypes matured and were harvested manually. Total As concentration in grain samples were determined using inductively coupled plasma mass spectrometry (ICP‐MS) following total sample digestion in acid, as described by Norton et al. (2014). Each unique combination of location and year was treated as an independent experiment, resulting in a total of five experiments conducted across four locations.

### 2.3 Single‐Experiment GWA Mapping

GWA mapping was performed using the PIQUE pipeline, which utilizes EMMAX to conduct the analyses on each phenotype (Norton et al. 2018). The mean grain As value from the original data in each experiment was log_10_ transformed to ensure a normal distribution and transformed values were used for subsequent GWA analysis. GWA mapping was conducted using 3.5M filtered SNPs and an efficient mixed model (EMMA) to control for population structure (PCA based on the first four principal components) and kinship (using IBS). A significance threshold of *p* < 0.0001 was used to determine significant SNPs as described in Norton et al. (2018). The false discovery rate (FDR) of detected associations was controlled for using Benjamini–Hochberg adjusted probabilities (Benjamini and Hochberg 1995). A significance threshold of 5% FDR was used to identify SNP associations (McCouch et al. 2016).

### 2.4 Multi‐Experiment GWA Mapping

A multi‐experiment analysis of GWA mapping for grain As concentration across the five field experiments in RDP1 was conducted using the mvLMM in GEMMA version 0.98 (Zhou and Stephens 2014). The log_10_ mean grain As concentration for each field experiment was converted to a *z*‐score by mean centering and dividing by the standard deviation. Trait data were analyzed for linkage against the 3.5M filtered SNPs. The mvLMM accounts for both population structure and relatedness among samples to control for potential confounding factors. The eigenvectors of the first four principal components were calculated using the smartpca program in EIGENSOFT (Patterson et al. 2006) and included in the model as fixed effects. The null hypothesis is that the effect of a single SNP across all experiments is zero, whereas the alternative hypothesis is a nonzero effect of at least one SNP tested using a Wald test (Ruang‐areerate et al. 2020). *p* values of all association tests are presented as Manhattan plots and observed *p* values against expected *p* values are presented by Q‐Q plots using the qqman package (Turner 2014). A significance threshold of *p* < 0.0001 was used to identify significant SNPs as described in Norton et al. (2018). SNPs were also tested to a 5% FDR based on the Benjamini–Hochberg procedure (Benjamini and Hochberg 1995).

### 2.5 Multi‐Locus GWA Mapping

Arsenic concentrations from each experiment (log_10_ transformed) were standardized using *z*‐score by mean centering and dividing by the standard deviation. The mean values of five standardized As traits were used to calculate a single mean phenotype value for multi‐locus GWA. To make sure the analysis was balanced, only the 253 genotypes that had As data in all five locations were used. The first four principal components were included in the GWA and the critical logarithm of odds (LOD) threshold was set at ≥ 3. Six multi‐locus GWAS methods (mrMLM, FASTmrMLM, FASTmrEMMA, pLARmEB, pKWmEB, and ISIS EM‐BLASSO) were performed using the MrMLM 4.0 package (Y.W. Zhang et al. 2020) in R Version 4.1.2s. Significant quantitative trait nucleotides (QTNs) for grain As were reported in the multi‐locus GWA mapping.

### 2.6 Clump Analysis of Significant SNPs

Following analysis using EMMAX and GEMMA, results were grouped across analyses based on empirical estimates of linkage disequilibrium (LD) between SNPs using CLUMP via the PLINK command “‐‐clump‐p1 0.0001 ‐‐clump‐p2 0.0001 ‐‐clump‐r2 0.5 ‐‐clump‐kb 500” as described in Ruang‐areerate et al. (2020). The ‐‐clump‐kb option sets the physical distance for clumping and was set to the average LD‐decay (500 kb) of all accessions in the RDP1 (K. Zhao et al. 2011). Clump QTLs with more than one significant SNP were reported in the single‐locus GWA mapping. When the positions of peak SNPs for QTLs across different field sites or GWAS methods overlapped or were within 500 kb of each other, these QTLs were regarded as the same QTLs identified across multiple field sites or GWAS methods.

### 2.7 Haplotype Analysis

The notable QTLs that were associated with grain As concentration in multiple locations and/or detected from different GWA methods were further investigated. The SNPs within 500 kb of peak SNPs of QTLs were extracted via plink, and LD heatmaps of these SNPs were constructed using the R package “LDheatmap.” The SNPs that were significantly associated (*p* < 0.01) with grain As concentration were extracted using PLINK, and nonsynonymous and synonymous SNPs within the genes were identified based on the gene models of the annotated Nipponbare reference genome from RGAP using the ANNOVAR software (K. Wang et al. 2010). The haplotypes based on visualization of the SNPs in the exon of the candidate genes in these notable QTLs were determined, and the phenotypic response for the cultivars with each haplotype was analyzed. The pairwise Wilcox test was used to determine the significant difference between Haplotypes I and II of candidate gene *OsPIP2;4*. Tukey's honestly significant difference (HSD) test was used to assess the significant difference among haplotypes of candidate gene *OsABCC1*.

### 2.8 Statistical Analysis

Boxplots and Pearson correlation analyses of grain As concentrations in different experiments were performed using the R packages “ggplot2” (Wickham 2016) and “corrplot” (Wei et al. 2021). Plots showing locations of QTLs and candidate genes across the 12 rice chromosomes were generated using the R packages “plotrix” (Lemon 2006) and “shape” (Wickham 2007).

## 3 Results

### 3.1 Grain Arsenic Concentrations Under Different Field Experiments

**FIGURE 1:**
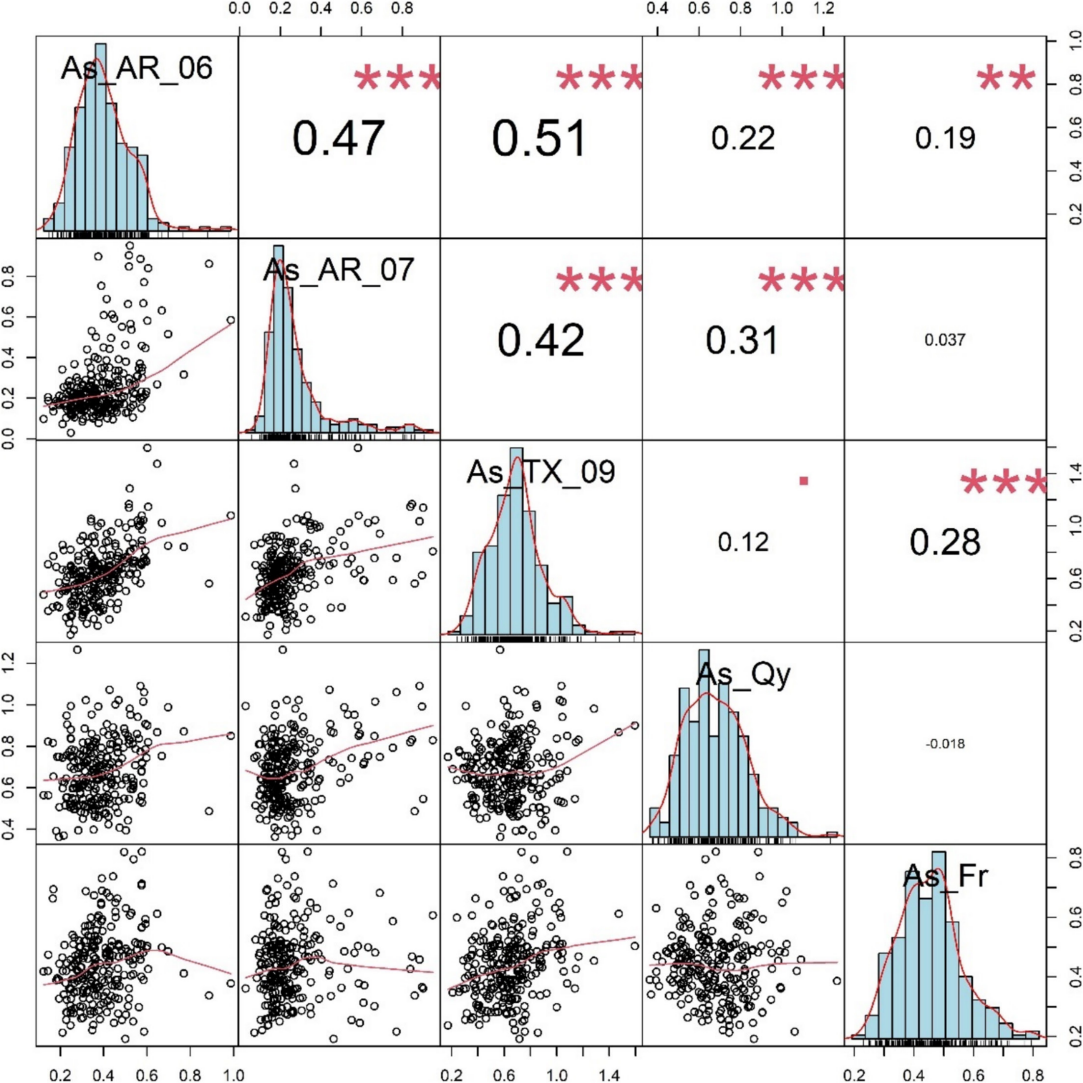
Relationship between grain arsenic in different fields. As_AR_06 and As_AR_07, grain arsenic in Arkansas in year 2006 and year 2007, respectively. As_TX_09, grain arsenic in Texas in the year 2009. As_Qy, grain arsenic at Qiyang in China. As_Fr, grain arsenic at Faridpur in Bangladesh. The lower‐left panels display scatters with the linear regression statistics between two traits, the diagonal histogram represents the distribution of As within population at different experiment sites, and the numbers in the upper‐right panels represent the correlation coefficient (positive numbers represent positive correlation, and negative numbers represent negative correlation). The asterisks represent significance: * stands for a *p*‐value less than 0.05; ** stands for a *p*‐value less than 0.01; *** stands for a *p*‐value less than 0.001.

The phenotypic data used in this study were collected and analyzed by Norton et al. (2014). The grain As concentrations of the RDP1 genotypes were significantly different across the four field sites. Additionally, at each site, the grain As concentrations differed significantly between the rice subpopulations (Norton et al. 2014). Grain As was also significantly correlated between the two Arkansas harvests (2006 and 2007), with *r* = 0.407, *p* < 0.001 (Norton et al. 2014). Here, we expand that analysis to include correlations across all five years and location experiments. Significant positive correlations between grain As from the three US field experiments at Arkansas in 2006 and 2007 and at Texas in 2009 were observed (*p* < 0.001, *r* = 0.42–0.51). The grain As from the experiment at Faridpur in Bangladesh correlated significantly with grain As in Arkansas in 2006 (*p* < 0.01, *r* = 0.19) and grain As in Texas (*p* < 0.001, *r* = 0.28). The grain As from the experiment at Qiyang in China significantly correlated with grain As in Arkansas in 2006 (*p* < 0.001, *r* = 0.22) and 2007 (*p* < 0.001, *r* = 0.31) (Figure 1).

### 3.2 Grain As QTLs Detected by Single‐Experiment GWA Mapping

**FIGURE 2:**
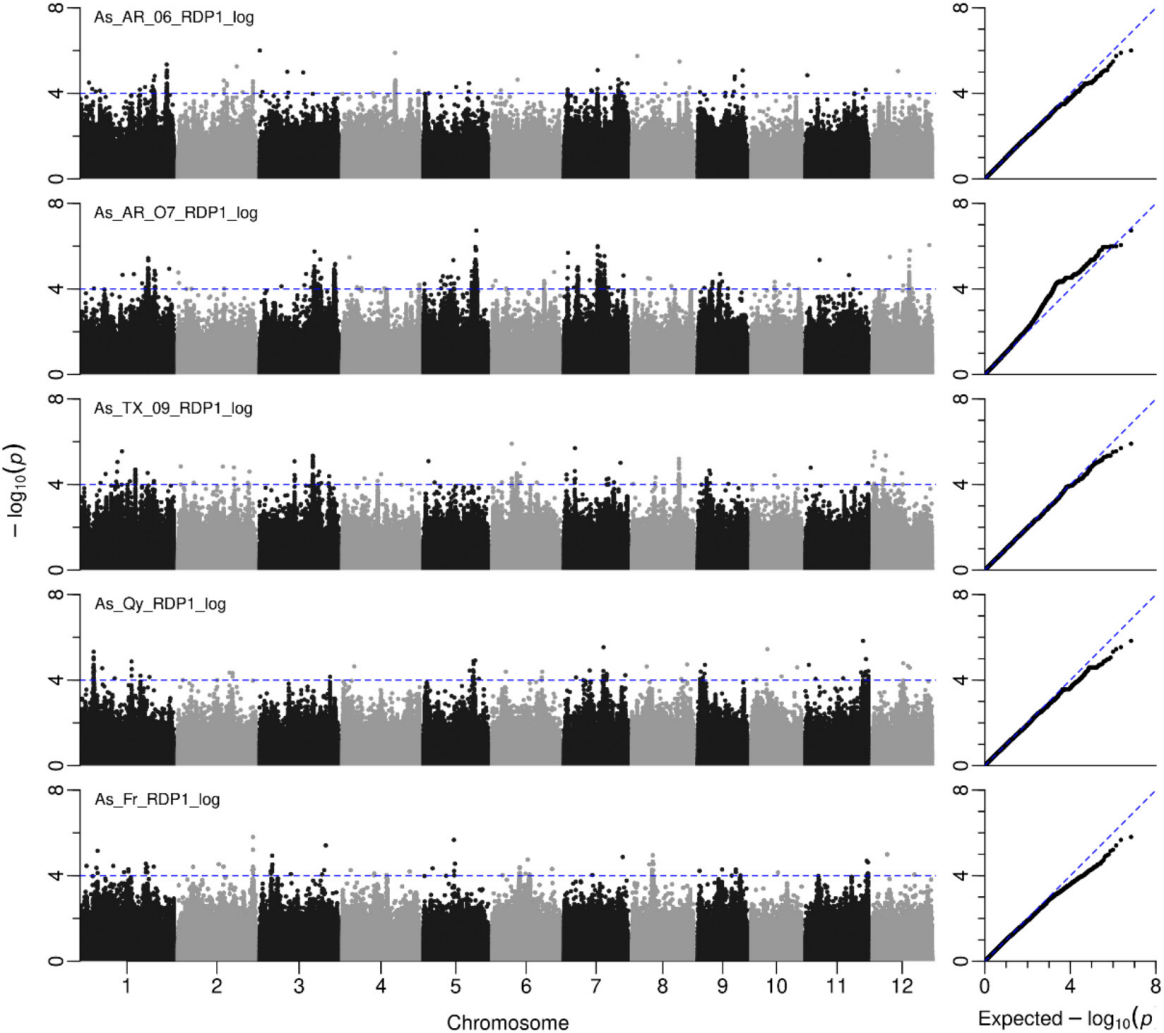
Genome‐wide association mapping for grain As concentration in RDP1 by using single‐experiment analysis. As_AR_06 and As_AR_07, grain arsenic in Arkansas in the year 2006 and year 2007, respectively; As_TX_09, grain arsenic in Texas in the year 2009; As_Qy, grain arsenic at Qiyang in China; As_Fr, grain arsenic at Faridpur in Bangladesh.

GWA mapping was conducted separately in five field experiments using EMMAX with 3.5M filtered SNPs (Figure 2). A total of 14, 31, 21, 10, and 14 QTLs were significantly associated with grain As concentration (−log_10_ *p* > 4) in Arkansas (2006), Arkansas (2007), Texas, Faridpur, and Qiyang, respectively (Table S2 and Figure 2). Of these, 48 QTLs exhibited an effect size of 10% or greater (Table S2). The most statistically significant QTLs (−log_10_ *p* > 5.95, *p = p* value of the peak SNP per QTL) were identified on Chromosome 5 at ~23.95 Mb (−log_10_ *p* = 5.96) with a total of 534 significant SNPs, on Chromosome 5 at ~24.39 Mb (−log_10_ *p* = 6.73) with a total of 135 significant SNPs, and on Chromosome 7 at ~15.56 Mb (−log_10_ *p* = 5.99) with a total of 173 significant SNPs. As the effect sizes for the QTLs on Chromosome 5 at ~23.95 and ~24.39 Mb were opposite (17.1% and −15.6%, respectively) and the MAFs were different for each marker (11.9% and 24.7%, respectively), these QTLs were treated separately. The three QTLs with the highest ‐log_10_ *p* were all identified from analysis of Arkansas 2007 data alone. A total of seven QTLs were detected across more than one field experiment (Table S2). A QTL was determined to be in the same location when the positions of peak SNPs for QTLs across different field sites overlapped or were within 500 kb of each other. One collocated QTL was detected on each of Chromosomes 2, 3, and 5, and two each on Chromosomes 1 and 7. It is notable that the QTL identified on Chromosome 1 ranging from 33.94 to 34.32 Mb was detected in three field experiments: Arkansas in year 2006, Arkansas in year 2007, and Texas in year 2009.

### 3.3 Grain As QTLs Detected by Multi‐Experiment GWA Mapping

**FIGURE 3:**
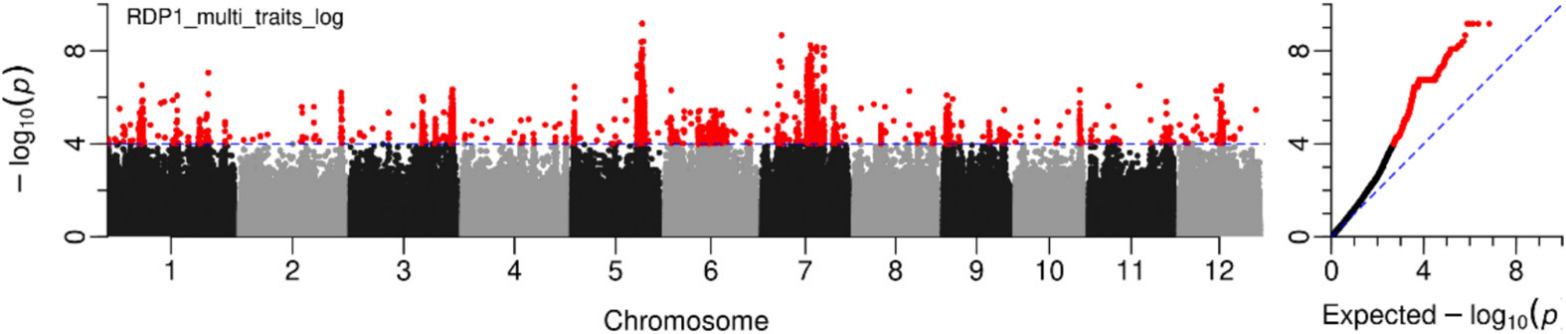
Genome‐wide association mapping for grain As concentration in RDP1 by using multi‐experiment analysis. SNPs were highlighted in red color when passed to a 5% FDR using Benjamini–Hochberg–adjusted probabilities.

GWA mapping for grain As concentration (*z*‐score after transformation) was performed using GEMMA on the 253 accessions that were common across all five field experiments (Figure 3). A total of 111 QTLs were significantly associated with grain As concentration (−log_10_ *p* > 4), with at least one SNP in each QTL passing the 5% FDR threshold (Table S3, Figure 3). Six of these QTLs were notable based on statistical significance (−log_10_ *p* > 8, *p = p* value of the peak SNP per QTL). They were located on Chromosome 5 at ~23.95 Mb with 1370 significant SNPs (−log_10_ *p* of peak SNP = 9.17), on Chromosome 5 at ~24.38 Mb with 352 significant SNPs (−log_10_ *p* = 8.41), on Chromosome 7 at ~6.73 Mb with two significant SNPs (−log_10_ *p* = 8.67), on Chromosome 7 at ~16.58 Mb with 619 significant SNPs (−log_10_ *p* = 8.24), on Chromosome 7 at ~18.71 Mb with 197 significant SNPs (−log_10_ *p* = 8.16), and on Chromosome 7 at ~21.12 Mb with 55 significant SNPs (−log_10_ *p* = 8.13).

Of the 111 QTLs detected by the multi‐experiment GWA mapping, 40 QTLs were consistent with those identified based on single‐experiment GWA mapping (Table S4 and Figures 2 and 3). For example, the QTL on Chromosome 1 ranging from 33.84 to 34.37 Mb was co‐detected by three independent field experiments: Arkansas in 2006 and 2007 and Texas in 2009. The QTL on Chromosome 5 at ~23.95 Mb was also identified in the single experiment, Arkansas in year 2007. The QTL on Chromosome 7 at ~15.5 Mb with a total of 510 significant SNPs was co‐detected by the single‐experiment GWA mapping in Arkansas in the years 2006 and 2007. The QTL on Chromosome 9 at ~1.35 Mb with 1074 significant SNPs was also identified in the single‐experiment GWA mapping in Qiyang with two significant SNPs.

A total of 71 QTLs for grain As that were identified using multi‐experiment GWA analysis were not detected by single‐experiment analysis (Table S4), for example, the QTL on Chromosome 1 at ~11.74 Mb with 181 significant SNPs, the QTL on Chromosome 5 at ~0.89 Mb with a total of 65 significant SNPs, and the QTL on Chromosome 12 at ~14.45 Mb with a total of 151 significant SNPs (Table S4 and Figures 2 and 3).

### 3.4 Grain As QTNs Detected by Multi‐Locus GWAS Methods

When the mean value of the five standardized As phenotypic data was used to conduct the multi‐locus GWA with the six methods in the mrMLM package in R, a combined total of 72 significant QTNs were identified (Table 1). The FASTmrMLM (23) methods identified the most QTNs (23), followed by pLARmEB (21), pKWmEB (19), mrMLM (12), and ISIS EM‐BLASSO (11), while the FASTmrEMMA method identified the lowest number of QTNs (2). The five QTNs notable for having −log_10_ *p* > 12 were SNP_1_30876860 (−log_10_ *p* = 12.5) explaining 5.53% of phenotypic variation, SNP_1_31421621 (−log_10_ *p* = 15.7) explaining 41.8% of phenotypic variation, SNP_3_24636405 (−log_10_ *p* = 12.5) explaining 11.56% of phenotypic variation, SNP_7_25050720 (−log_10_ *p* = 14.9) explaining 7.86% of phenotypic variation, and SNP_12_13859027 (−log_10_ *p* = 12.5) explaining 3.93% of phenotypic variation (Table 1).

**TABLE 1:** Significant QTNs for grain As concentration detected by multi‐locus methods.

SNP	Chr	Marker position (bp)	QTN effect	−log_10_(P)	*r* ^2^ (%)^a^	Methods^b^
SNP_1_2089929	1	2,089,929	−0.07	3.90	2.45	5
SNP_1_11876654	1	11,876,654	0.08	6.47	3.28	5
SNP_1_11955697	1	11,955,697	−0.10	6.48	2.37	2
SNP_1_13379811	1	13,379,811	0.13	4.72	2.58	4
SNP_1_13975014	1	13,975,014	0.13	5.07	2.33	3
SNP_1_28484917	1	28,484,917	0.09, 013	4.37, 6.92	1.37, 4.19	2, 6
SNP_1_28959591	1	28,959,591	−0.18	5.71	4.95	4
SNP_1_29463236	1	29,463,236	−0.14	8.87	6.48	5
SNP_1_30876860	1	30,876,860	0.20	12.55	5.53	2
SNP_1_30877239	1	30,877,239	0.20	3.72	3.00	4
SNP_1_31421621	1	31,421,621	0.52	15.67	41.80	4
SNP_1_31536782	1	31,536,782	0.22	10.26	11.44	2
SNP_1_34323683	1	34,323,683	−0.11	7.25	3.03	2
SNP_2_6906642	2	6,906,642	0.09	3.78	2.28	2
SNP_2_7398675	2	7,398,675	−0.13	5.06	2.15	3
SNP_2_9450368	2	9,450,368	0.14	5.68	0.76	6
SNP_2_13001295	2	13,001,295	0.17	4.05	2.30	5
SNP_2_19542765	2	19,542,765	−0.05	3.77	0.31	6
SNP_2_33277520	2	33,277,520	0.09, 0.10	4.27, 5.33	1.26, 3.15	2, 5
SNP_2_34564546	2	34,564,546	0.17	6.32	2.53	6
SNP_2_34591582	2	34,591,582	−0.33, −0.19	6.53, 11.94	3.79, 6.35	3, 5
SNP_2_34977775	2	34,977,775	0.17	4.00	2.82	4
SNP_3_4919439	3	4,919,439	−0.12	6.93	1.38	2
SNP_3_11299789	3	11,299,789	0.19	4.41	3.65	4
SNP_3_13056272	3	13,056,272	−0.15	6.30	3.41	3
SNP_3_13447204	3	13,447,204	−0.14	6.78	2.51	6
SNP_3_24636405	3	24,636,405	−0.18, −0.14	5.89, 12.45	2.54, 11.56	5, 6
SNP_3_29244009	3	29,244,009	0.33	3.83	4.32	1
SNP_4_6190448	4	6,190,448	−0.10	4.92	2.04	3
SNP_4_6488350	4	6,488,350	0.10, 0.13	5.58, 8.43	2.06, 2.46	2, 6
SNP_4_16168608	4	16,168,608	0.07–0.28	3.74–8.40	1.50–4.47	1, 2, 5
SNP_4_16170098	4	16,170,098	0.11	6.80	1.87	6
SNP_4_23433069	4	23,433,069	0.12	6.50	3.21	5
SNP_4_23981762	4	23,981,762	0.17	6.36	3.12	4
SNP_4_31803015	4	31,803,015	0.09	4.01	0.62	2
SNP_4_32081836	4	32,081,836	−0.05	4.27	1.54	5
SNP_4_33137021	4	33,137,021	−0.14	6.92	1.43	2
SNP_5_1982590	5	1,982,590	0.21	8.60	5.67	4
SNP_5_2207493	5	2,207,493	0.18	9.12	4.91	5
SNP_5_9134248	5	9,134,248	−0.14	5.66	2.26	5
SNP_5_14757146	5	14,757,146	−0.16	3.92	3.72	4
SNP_5_15728722	5	15,728,722	−0.22	7.40	2.30	4
SNP_6_1491837	6	1,491,837	0.13	6.49	2.87	3
SNP_6_4806530	6	4,806,530	0.11	4.27	2.15	5
SNP_6_23051425	6	23,051,425	−0.12	4.96	2.04	5
SNP_7_7814446	7	7,814,446	0.08, 0.10	3.90, 6.89	0.87, 2.08	2, 6
SNP_7_8168133	7	8,168,133	−0.06	4.13	1.70	5
SNP_7_10448596	7	10,448,596	0.05	4.26	0.32	6
SNP_7_15970751	7	15,970,751	−0.27	8.09	6.51	4
SNP_7_16105614	7	16,105,614	0.11, 0.11	4.75, 5.09	0.77, 1.03	2, 6
SNP_7_17198761	7	17,198,761	0.24, 0.25	7.04, 7.78	3.65, 5.80	3, 5
SNP_7_19392618	7	19,392,618	0.15	6.87	1.02	6
SNP_7_25050720	7	25,050,720	−0.22 to −0.08	3.72–14.89	0.50–7.86	2, 3, 4, 5, 6
SNP_7_26120188	7	26,120,188	0.15	6.85	2.18	2
SNP_7_27379415	7	27,379,415	0.16	7.95	2.00	2
SNP_8_15994461	8	15,994,461	0.19, 0.25	7.58, 7.75	2.86, 3.33	3, 5
SNP_8_21207866	8	21,207,866	0.15	5.13	1.58	3
SNP_8_26869785	8	26,869,785	0.19	5.35	1.92	3
SNP_9_12628970	9	12,628,970	0.19, 0.22	8.03, 11.93	2.56, 2.65	2, 6
SNP_9_14359329	9	14,359,329	0.17	8.82	3.65	5
SNP_10_1274853	10	1,274,853	−0.05	3.77	0.21	6
SNP_10_11757992	10	11,757,992	0.08	6.36	1.09	2
SNP_10_14875442	10	14,875,442	−0.15	9.86	3.61	6
SNP_11_5489458	11	5489458	0.11	6.00	1.73	6
SNP_11_18003112	11	18,003,112	0.08	3.89	0.45	2
SNP_11_21472458	11	21,472,458	0.11	8.15	1.54	2
SNP_11_22469803	11	22,469,803	0.18	10.54	4.66	6
SNP_11_22976960	11	22,976,960	−0.12	7.23	1.04	6
SNP_11_23730796	11	23730796	0.10	5.14	1.43	2
SNP_11_27538579	11	27538579	0.07	4.46	0.49	6
SNP_12_13859027	12	13859027	−0.17	12.54	3.93	6
SNP_12_14597524	12	14597524	0.17	6.92	1.75	2

^a^ Phenotypic variation explained.

^b^ 1: FASTmrEMMA; 2: FASTmrMLM; 3: ISIS EM‐BLASSO; 4: mrMLM; 5: pKWmEB; 6: pLARmEB.

A comparison of the QTNs detected by different multi‐locus methods identified that 12 QTNs were co‐detected by more than one method. These QTNs were located with one each on Chromosomes 1, 3, 8, and 9, two each on Chromosomes 2 and 4, and four each on Chromosome 7. The QTN SNP_7_25050720 was detected by five multi‐locus methods (pLARmEB, ISIS EM‐BLASSO, FASTmrMLM, mrMLM, and pKWmEB), and the QTN SNP_4_16168608 was detected by three multi‐locus methods (FASTmrEMMA, FASTmrMLM, and pKWmEB) (Table 1).

**FIGURE 4:**
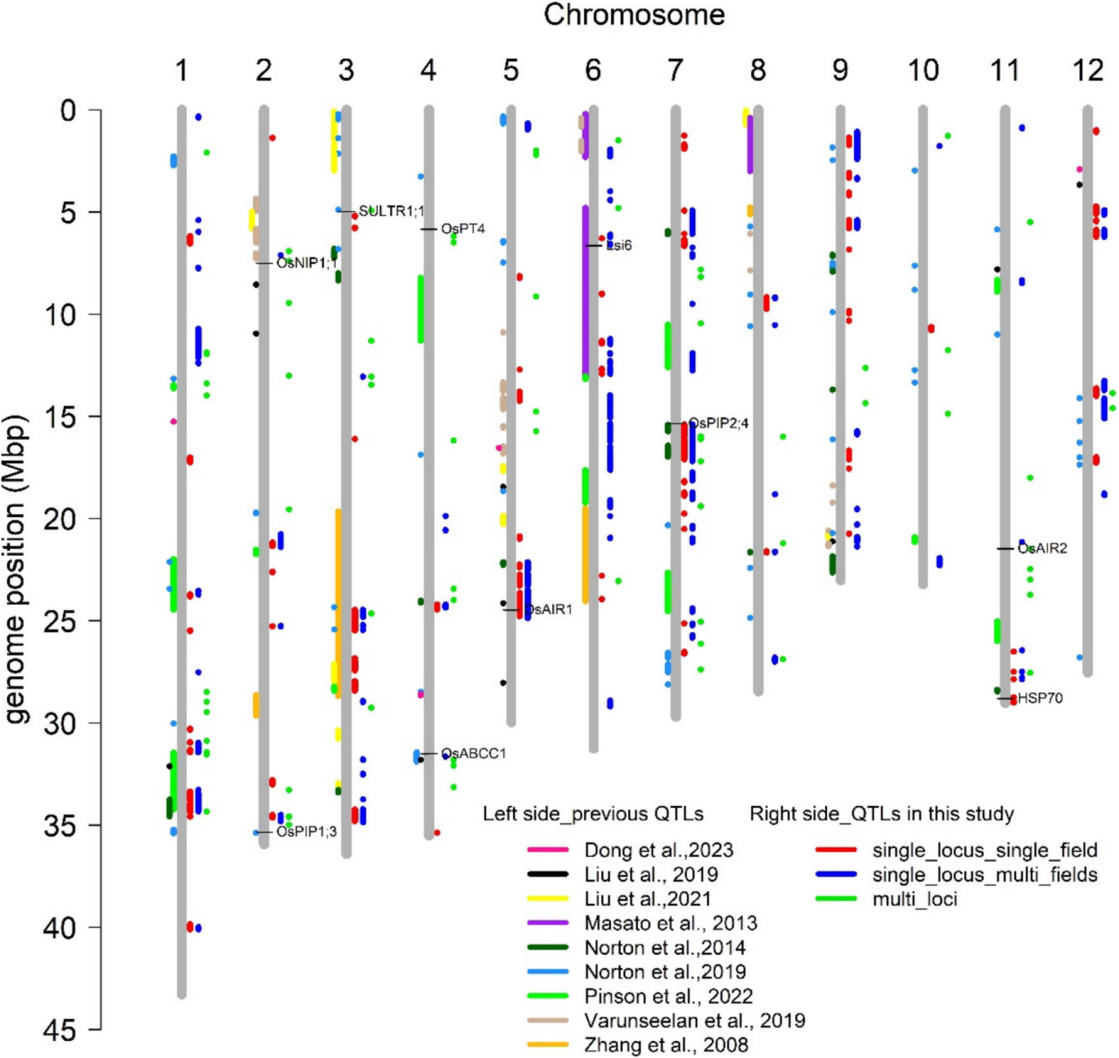
Locations of QTLs for As detected in this study and previous studies. Candidate genes identified in this study were indicated by horizontal lines.

A comparison between QTNs identified using multi‐locus GWA methods and QTLs detected through single‐locus GWA methods revealed that 21 of the 72 QTNs were colocated with QTLs (Table S4 and Figure 4). For example, three QTNs (SNP_2_34564546, SNP_2_34591582, and SNP_2_3497775) were found in the QTL on Chromosome 2 spanning 34.53–34.82 Mb, which was detected by single‐locus GWAS methods (both EMMAX and GEMMA). The QTN SNP_7_15970751, detected via the multi‐locus mrMLM method, was located within the QTL on Chromosome 7, ranging from 15.39 to 16.07 Mb, which was also detected by single‐locus GWAS methods.

### 3.5 QTL on Chromosome 7 at ~15.55 Mb

The QTL on Chromosome 7 from 15.39 to 16.07 Mb was detected using both the single‐experiment and multi‐experiment GWA methods and also detected by multi‐locus GWAS. The effect size of peak SNP (SNP_7_15551740) for this QTL identified by the single‐experiment GWA is 23.7% (Table S2). A significant QTN (SNP_7_15970751) was co‐detected in this QTL by multi‐locus GWAS; this QTN effect is 27%, and this QTN explains 6.51% of phenotypic variation. Four genes annotated as aquaporin proteins are found in this QTL region, namely, *OsPIP2;4* (LOC_Os07g26630), *OsPIP2;9* (LOC_Os07g26640), *OsPIP2;5* (LOC_Os07g26660), and *OsPIP2;1* (LOC_Os07g26690). Due to their close proximity, these genes are linked within the studied population. *OsPIP2;4* (positioned at 15,358,960–15,360,495 bp) was selected as the gene to report as a candidate for this QTL based on a priori knowledge about its function. The transcript level of *OsPIP2;4* in rice roots and shoots has been reported to be significantly downregulated in response to As(III) treatment, while overexpression of *OsPIP2;4* in *Arabidopsis thaliana* (Arabidopsis) yielded enhanced As(III) tolerance, and in long‐term uptake experiments, these transgenic plants showed no significant accumulation of As (Mosa et al. 2012). The study of Mosa et al. (2012) did not explore the expression of the other three PIP genes in this location (*OsPIP2;1*, *OsPIP2;5*, and *OsPIP2;9*); however, in a previous analysis in rice roots exposed to arsenate, none of these genes where either upregulated or downregulated (Norton et al. 2008).

**FIGURE 5:**
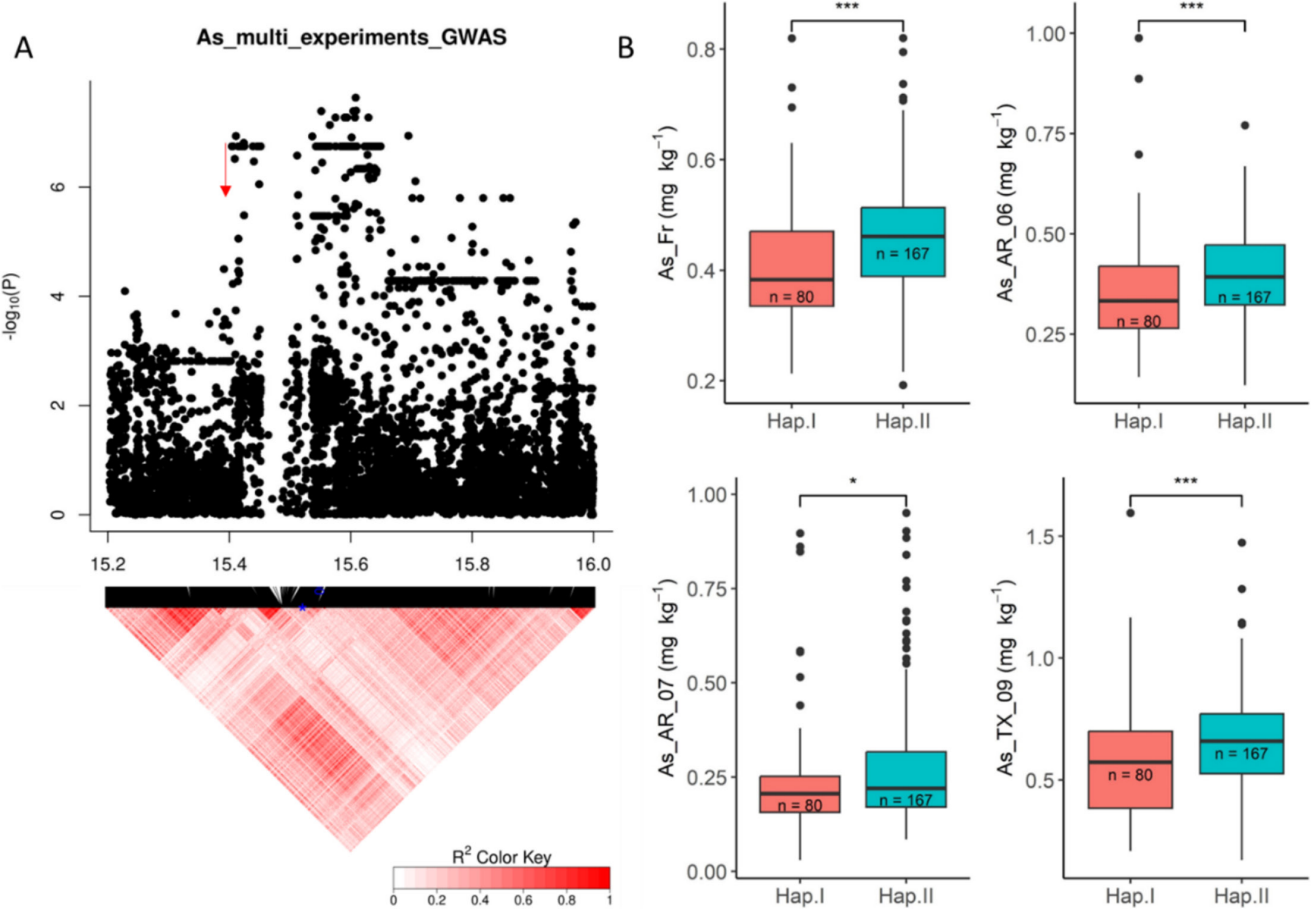
The QTL for As concentration on Chromosome 7 at 15.5 Mb. (A) Local Manhattan plot (top) and LD heat map (bottom) of QTL on Chromosome 7; the arrow indicates the position of candidate gene *OsPIP2;4* (LOC_Os07g26630, positioned at 15,358,960–15,360,495 bp). (B) As concentration for indicated haplotypes of *OsPIP2;4* at Faridpur in Bangladesh, Arkansas (2006 and 2007) and Texas in the United States. Asterisks represent significance: * stands for a *p*‐value less than 0.05; ** stands for a *p*‐value less than 0.01; *** stands for a *p*‐value less than 0.001.

A total of 42 SNPs were identified in *OsPIP2;4* in the 253 RDP1 used in this study and 12 SNPs were in the exons of this gene, three of which were nonsynonymous SNPs. The physical positions of these three nonsynonymous SNPs from the beginning of the chromosome were at 15,359,866 bp (T/C polymorphism), 15,359,874 bp (G/A polymorphism), and 15,360,078 bp (G/A polymorphism), which resulted in the following amino acid changes: V237A, V240I, and V270I, respectively. The cultivars in this study carried two haplotypes for these 12 SNPs: I (*n* = 80) and II (*n* = 167) (Table S5). The cultivars carrying Haplotype I (mean As concentration: 0.41, 0.36, 0.24, and 0.58 mg kg^−1^ for plants growing in Faridpur, Arkansas in 2006 and 2007, and Texas, respectively) had significantly (*p* < 0.05) lower grain As concentration compared to those carrying Haplotype II (mean As concentration: 0.46, 0.40, 0.28, and 0.68 mg kg^−1^ for plants growing in Faridpur, Arkansas in 2006 and 2007, and Texas, respectively) across four of the five field studies, resulting in a reduction in grain As of 11.78%–15.78% (Figure 5). Only the grains harvested from plants at the Qiyang location did not exhibit a statistical difference in As concentration between the two haplotypes. Among the rice subpopulations, the *temperate japonicas* have, as a group, the lowest grain As (Norton et al. 2014; Pinson et al. 2015), and Haplotype I, associated with reduced grain As, is the predominant haplotype among the *temperate japonica* cultivars (83%) (Table S5 and Figure S1a). While Haplotype I is completely absent among the *indica* RDP1 cultivars, all other subpopulations had five or more cultivars carrying Haplotype I that can be used by breeders as gene donors.

As already mentioned, the four *OsPIP* genes in this region are in close physical proximity to each other; therefore, while the haplotype analysis of *OsPIP2;4* has been presented, it is important to note that the cultivars that have the low grain arsenic haplotype, Haplotype I, of *OsPIP2;4* also have a low grain arsenic haplotype in the other three *OsPIP* genes in this region. Of the 81 cultivars identified as having the low grain haplotype, Haplotype I, for *OsPIP2;4*, 76, 77, and 68 of these cultivars also have a low grain arsenic haplotypes for *OsPIP2;9*, *OsPIP2;5*, and *OsPIP2;1*, respectively. These haplotypes are Haplotype I for *OsPIP2;9*, Haplotype V for *OsPIP2;5*, and Haplotype IV for *OsPIP2;1* (Figures 5 and S2 and Table S5).

### 3.6 QTN on Chromosome 4 at ~31,803,015 bp

**FIGURE 6:**
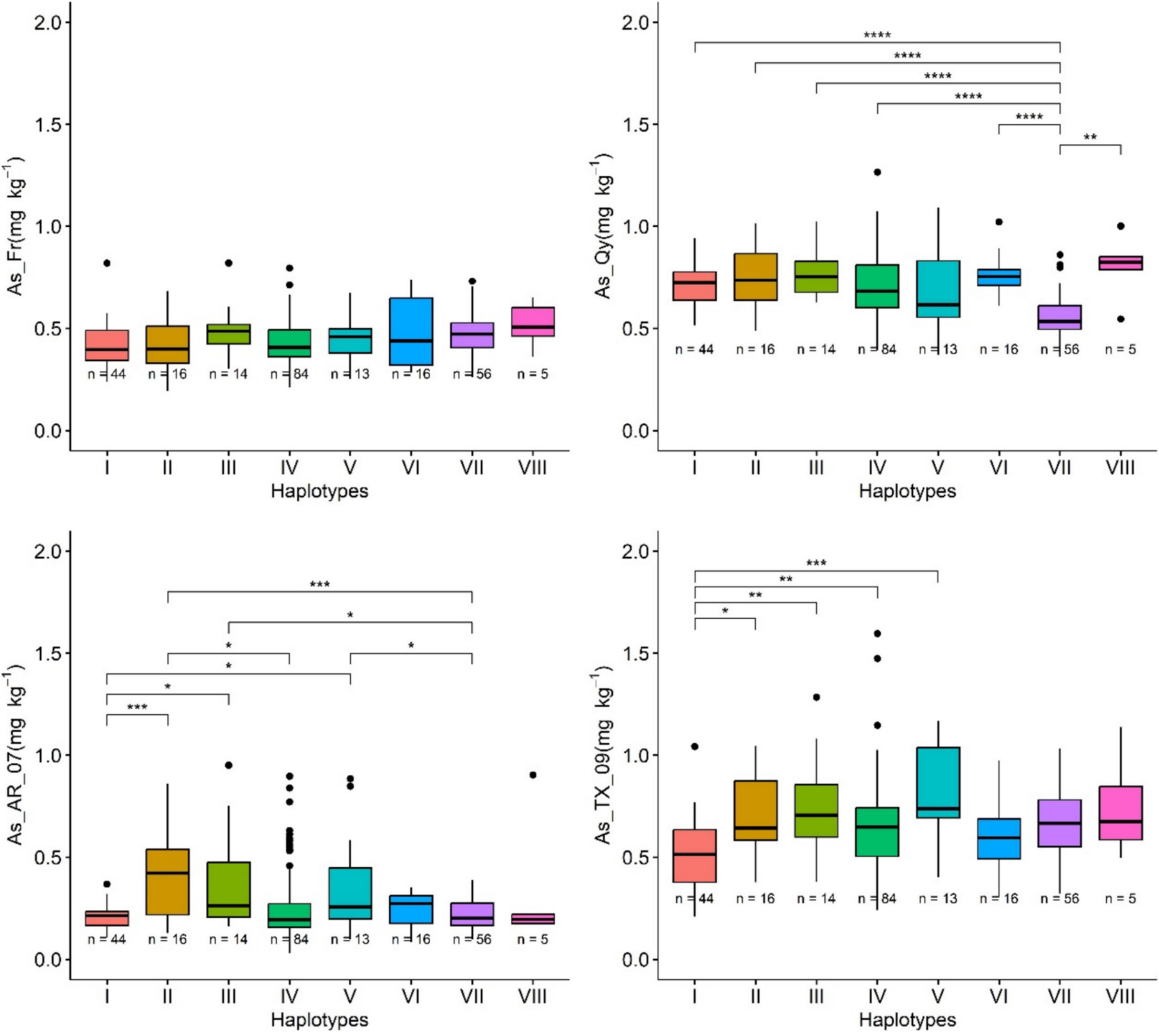
As concentration for indicated haplotypes of *OsABCC1* at Faridpur in Bangladesh, Qiyang in China, and Arkansas and Texas in the United States. The asterisks represent the significance of mean differences: * stands for a *p*‐value less than 0.05; ** stands for a *p*‐value less than 0.01; *** stands for a *p*‐value less than 0.001.

The QTN SNP_4_31803015 was detected by the multi‐locus GWA method (FASTmrMLM), explaining 0.62% of phenotypic variation, and a QTL (on Chromosome 4 at ~31.64 Mb) close to this QTN was detected using a single‐locus method (multi‐experiment GWA by GEMMA). The gene *OsABCC1* (positioned at 31,502,940–31,514,308 bp), which is 0.29 kb from this QTN, was considered as a candidate gene, as the ABC transporter *OsABCC1* has been reported to sequester As into the vacuoles of phloem companion cells in stem nodes and thus reduce As accumulation in rice grain (Song et al. 2014). A total of 55 SNPs were found in this candidate gene from the 253 cultivars, of which seven were nonsynonymous SNPs. The physical positions of these seven SNPs were at 31,507,580 (C/G polymorphism), 31,513,040 (C/T polymorphism), 31,513,380 bp (G/A polymorphism), 31,514,034 bp (G/A polymorphism), 31,514,212 bp (T/A polymorphism), 31,514,242 bp (T/A polymorphism), and 31,514,248 bp (C/A polymorphism), which result in the following amino acid changes: V814L, R276Q, P206L, A92V, T33S, N23Y, and V21L, respectively. The cultivars in this study carried eight haplotypes (*n* ≥ 5) for these seven SNPs (Table S6): I (*n* = 44), II (*n* = 16), III (*n* = 14), IV (*n* = 84), V (*n* = 13), VI (*n* = 16), VII (*n* = 56), and VIII (*n* = 5) (Figure 6). The cultivars that had Haplotype VII (mean = 0.56 mg kg^−1^) showed significantly lower grain As concentration (*p* < 0.01) than other haplotypes when the plants were grown in the fields at Qiyang, China (means ranged from 0.68 to 0.80 mg kg^−1^ for Haplotypes I–VI and VIII), resulting in a reduction in grain arsenic concentration by 21.4%–44.8%. With cultivars of the *indica* subpopulation being preferred throughout most of China, it is important to note that none of the *indica* cultivars within RDP1 contain Haplotype VII, which was instead common among *tropical japonica* cultivars (Table S6 and Figure S1b). In contrast, the cultivars carrying Haplotypes I (mean = 0.21 mg kg^−1^) and VII (mean = 0.22 mg kg^−1^) had lower grain As concentrations than other haplotypes (mean ranged from 0.25 to 0.41 mg kg^−1^) when grown in the fields at Arkansas in 2007 and Texas 2009 (Figure 6). Rice varieties produced commercially in the US Mid‐South, including Arkansas and Texas, are predominantly of the *tropical japonica* subpopulation. While the more desirable Haplotype I was significantly more common among *temperate japonica* cultivars, two of the 67 *tropical japonica* cultivars in RDP1 do contain Haplotype I, including the historically grown US medium grain cultivar “Saturn” and the US long grain “C57‐5043.” In fact, C57‐5043 contains the haplotype associated with reduced grain As in the US Mid‐South at both the *OsABCC1* and *OsPIP2;4* genes.

It is especially interesting to note that the haplotype common among *tropical japonica* cultivars was associated with reduced grain As in the experiment conducted in China, where *indica* cultivars prevail, but was not the haplotype with lowest grain As in the experiments conducted in the US Mid‐South, where *tropical japonica* cultivars prevail. This might indicate that the various haplotypes/alleles have different impacts on inorganic versus organic As. The soil in Qiyang, China, is known to be relatively high in inorganic As (Norton et al. 2014), while the As in rice grains produced in the US Mid‐South is generally 50% or more organic As (Williams et al. 2005; F. Zhao et al. 2013).

## 4 Discussion

### 4.1 Comparison of Different GWAS Methods

In this study, eight GWAS methods, EMMAX, GEMMA, mrMLM, FASTmrMLM, FASTmrEMMA, pLARmEB, pKWmEB, and ISIS EM‐BLASSO, were used to identify grain As QTLs and QTNs. This approach has led to the identification of a large number of QTLs and QTNs for grain arsenic. EMMAX and GEMMA are single‐locus approaches while the others are multi‐locus approaches. EMMA has been a gold standard method for GWAS because it corrects for population structure and genetic relatedness in model organism association mapping, which can reduce false positives (Type I errors), but this approach is computationally impractical (Kang et al. 2008). Kang et al. (2010) modified EMMA and developed EMMAX whereby the estimation of variance components was approximated, therefore removing the requirement to estimate this for each marker. EMMAX has reduced computation time (Kang et al. 2010). GEMMA has been developed from the EMMA algorithm (Kang et al. 2008). The GEMMA model can fit both univariate linear mixed models and mvLMMs for testing association with a single trait and multiple traits, respectively (Zhou and Stephens 2014). However, most quantitative traits are controlled both by a few genes with major effects and by numerous polygenes with minor effects, which the single‐locus GWAS methods fail to detect. Multi‐locus GWAS methods were developed to overcome this issue, such as the mrMLM, FASTmrMLM, FASTmrEMMA, pLARmEB, pKWmEB, and ISIS EM‐BLASSO (X. Liu et al. 2016; Ren et al. 2018; Tamba et al. 2017; Tamba and Zhang 2018; J. Zhang et al. 2017). All six of these methods involve two‐step algorithms. During the first step, a single‐locus GWAS method is applied to scan the entire genome, and putative QTNs are detected according to a less stringent critical value, such as *p* < 0.005. During the second step, all selected putative QTNs are examined by a multi‐locus GWAS model to detect true QTNs (X. Liu et al. 2016; Ren et al. 2018; Tamba et al. 2017; Tamba and Zhang 2018; J. Zhang et al. 2017). Each of these methods has its own advantages and limitations. For example, FASTmrEMMA is more powerful in QTN detection and model fit, exhibits less bias in QTN effect estimation, and requires less running time, though its two‐step process complicates the determination of suitable thresholds for marker selection in the first step (Wen et al. 2018; Y. Xu et al. 2018). The LASSO method performs well for detecting large‐effect QTL but poorly for small‐effect QTL and fails to handle a large number of markers (Y. Xu et al. 2018). ISIS EM‐BLASSO reduced the number of markers via correlation learning to a moderate number and was then employed to select variables in the reduced model (Tamba et al. 2017). The results of this study were consistent with these reports, with the FASTmrEMMA detecting the most QTNs (23) and ISIS EM‐BLASSO detecting fewer QTNs (11). To improve the power of GWAS, several studies used different GWAS methods to mine target QTLs for complex agronomic traits (Cui et al. 2018; Y. Xu et al. 2018; Y.K. Zhang et al. 2015; Zhong et al. 2021), with the logic that QTLs detected by multiple methods may be more reliable.

### 4.2 Colocalization of QTLs for Arsenic Concentration Across Studies

This study employed two single‐locus GWAS methods (EMMAX and GEMMA) and six multi‐locus GWAS methods to identified QTLs and QTNs associated with grain As concentration using data from five field experiments (Figures 2 and 3 and Table 1). The phenotypic data used here had previously been analyzed for GWAS using EMMA, but only using a 44K SNP array (Norton et al. 2014). In that earlier study, significant associations were identified across the entire RDP1 population as well as within each subpopulation (*indica*, *aus*, *temperate japonica*, and *tropical japonica*). From the whole RDP1, a total of 15 QTLs were identified for grain As concentration when based on the criterion of the QTLs having two or more significant SNPs. In the current study, which utilized 3.5M filtered SNPs, 90 QTLs were detected using EMMAX, with nine QTLs overlapping between the two studies. The absence of six QTLs identified in the 2014 study but not detected here could be attributed to several factors. First, while the same phenotypic data were used, the number of genotypes analyzed differed; Norton et al. (2014) included all genotypes with data available for individual experiment, whereas the current study was limited to 253 genotypes that have As data across all field sites, potentially affecting QTL detection. Second, the current study employed data transformation, which is recommended for highly skewed datasets to reduce false positives. This transformation might have influenced the detection of certain QTLs near significance threshold. It is important to note that the most statistically significant QTLs remained unaffected by the data transformation.

QTLs and QTNs (± 5 kb, the average LD decay of RDP1) identified in this study were compared with those reported in the previously published studies. This comparison that 85 QTLs (and QTNs) identified here colocated with QTLs previously reported from the studies of various other populations (Dong et al. 2023; X. Liu et al. 2019; H. Liu et al. 2021; Masato et al. 2013; Norton et al. 2019; Pinson et al. 2022; Murugaiyan et al. 2019; J. Zhang et al. 2008) (Figure 4). Several colocalized QTLs and QTNs were particularly noteworthy due to their detection by multiple methods in this study and their previous identification in other studies. For example, the QTL located on Chromosome 3 at ~24.5 Mb, detected here by both single‐locus and multi‐locus GWA methods, was previously reported in two studies (Norton et al. 2019; J. Zhang et al. 2008), though it was not detected by prior analysis of this same phenotypic data (Norton et al. 2014). The QTL on Chromosome 9 at ~20.8 Mb was detected here by single‐locus (both EMMAX and GEMMA) GWAS and had been reported in three previous studies (H. Liu et al. 2021; Norton et al. 2019; Murugaiyan et al. 2019). The QTN located on Chromosome 4 at ~16.16 Mb, identified in this study by three multi‐locus methods (FASTmrEMMA, FASTmrMLM, and pKWmEB), was previously reported by Norton et al. (2019). These results indicate that there are environmentally stable QTLs for grain As and the underlying genes could be important for breeding low grain As cultivars.

### 4.3 Candidate Genes for As

In this study, 10 candidate genes previously reported to be involved with As stress in rice were located within 500 kb of peak SNPs and/or QTNs identified (Table S7 and Figure 4). The candidate gene *OsABCC1*, a rice C‐type ATP‐binding cassette (ABC) transporter, was identified within the QTL on Chromosome 4 at ~31.64 Mb, close to the QTN SNP_4_31803015. *OsABCC1* has been reported to be involved in the detoxification and reduction of As in rice grains by sequestering As in the vacuoles of the phloem companion cells of the nodes (Song et al. 2014).

Two candidate genes, *OsNIP1;1* and *OsNIP2;2* (*OsLsi6*), encode NIPs. *OsNIP1;1* was found in the QTL on Chromosome 2 at ~7.1 Mb and a QTN (SNP_2_7398675), which colocated with the QTL spanning 7.02–7.28 Mb, by Murugaiyan et al. (2019). *OsNIP2;2* (*OsLsi6*) was found in the QTL on Chromosome 6 from 6.54 to 6.61 Mb, which was also reported by Masato et al. (2013). Overexpression of *OsNIP1;1* can restrict As(III) loading into the xylem, thereby reducing As accumulation in rice grain (Sun et al. 2018). *OsNIP2;2* (*Lsi6*) is a silicon transporter in rice, directing silicon to either the seed husk or the flag leaf (Yamaji and Jian 2009); it mediates As(III) transport and may contribute to As loading into rice grain (Lindsay and Maathuis 2017).

A PIP, *OsPIP1;3*, was also identified. *OsPIP1;3* was found in the QTN on Chromosome 2 at ~34.97 Mb, detected by the multi‐locus GWA method, which colocated with the QTL identified in the BAAP population (Norton et al. 2019). This gene has been demonstrated to be significantly downregulated in rice root and shoot under As(III) stress (Mosa et al. 2012). In addition to *OsPIP1;3*, a cluster of PIP genes on Chromosome 7 was identified in the QTL on Chromosome 7 between 15.39 and 16.01 Mb; this QTL was detected by both single‐locus (EMMAX and GEMMA) and multi‐locus (mrMLM) GWAS methods. This region is interesting as it contains four PIP genes in close proximity. Haplotype analysis of the PIP genes in this region showed that there was a low grain arsenic haplotype (made up of predominantly *japonica* cultivars) that was present across all four genes. Therefore, it is not possible to separate out which of these is the most likely candidate gene based on allelic variation. However, it has been demonstrated that *OsPIP2;4* was significantly downregulated in rice root and shoot under As(III) stress and that overexpression of *OsPIP2;4* in Arabidopsis resulted in enhanced As(III) tolerance, with transgenic plants showed no significant accumulation of As (Mosa et al. 2012). The Mosa et al. (2012) study did not report on the other PIPs in this cluster, and it is noteworthy that the transcriptomic study of rice root response to arsenate by Norton et al. (2008) did not reveal any of these aquaporins as arsenic responsive. Based on the information above, any or all of these PIPs should be considered as potentially contributing to arsenic uptake in rice.

*OsPT4* (*OsPht1;4*), a member of the Pht1 family of phosphate transporters, was identified as the candidate gene for a QTL on Chromosome 4 at ~6.19 Mb. The Pht1 family of genes encodes inorganic phosphate (Pi) transporters localized to the plasma membrane. These transports have been implicated in As uptake and transport in rice (Kamiya et al. 2013). Two candidate genes, *OsAIR1* and *OsAIR2*, are *Oryza sativa* arsenic‐induced RING E3 ligase genes. *OsAIR1* was the candidate gene for the QTL on Chromosome 5 ranging from 24.00 to 24.78 Mb, detected via EMMAX. *OsAIR2* was the candidate gene of the QTL (QTN) on Chromosome 11 at ~21.47 Mb, identified by both single‐locus and multi‐locus GWA methods. Both *OsAIR1* and *OsAIR2* were induced under As exposure (Hwang et al. 2017, 2016). Overexpression of *OsAIR1* in rice influenced seeding root length and cotyledon expansion under As(V) expression (Hwang et al. 2016). Heterogeneous overexpression of *OsAIR2* in Arabidopsis enhanced seed germination and increased root length under As(V) stress conditions (Hwang et al. 2017).

## 5 Conclusion

In this study, two single‐locus GWAS methods (EMMA and GEMMA) and six multi‐locus GWAS methods (FASTmrEMMA, FASTmrMLM, ISIS EM‐BLASSO, mrMLM, pKWmEB, and pLARmEB) were used to identify the loci associated with grain As accumulation across five field experiments. There were 90 and 111 QTLs for grain As detected by using the EMMAX and GEMMA, respectively. A total of 72 QTNs for grain As were detected from six multi‐locus GWAS methods. Of these, 21 QTLs (QTNs) were co‐detected by both single‐locus and multi‐locus GWAS methods. A total of 10 candidate genes were identified. The rice ABC transporter Os*ABCC1* was detected by GEMMA and FASTmrMLM GWAS methods. This approach of using an array of statistical methods and focusing on loci that appear in more than one study has the advantage of not being reliant on a single set of computational criteria. These QTLs, QTNs, and candidate genes represent promising targets for further genetic and breeding efforts aimed at reducing As accumulation in rice grain.

## Author Contributions

C.C., A.H.P., G.J.N.: conceptualization; C.C., P.R., A.J.T., a.d.: methodology; C.C., a.d., S.R.M.P., G.C.E., A.H.P., G.J.N.: formal analysis; D.E.S., S.R.M.P., G.C.E.: resources; C.C.: data curation; C.C.: writing – original draft; C.C., P.R., a.d., A.J.T., D.E.S., S.R.M.P., G.C.E., A.H.P., G.J.N.: writing – review & editing; C.C., G.C.E.: visualization.

## Conflicts of Interest

The authors declare no conflicts of interest.

## Supporting information


**Table S1** Phenotype data for the rice varieties grown at the five field experiments.
**Table S2.** Summary of QTLs identified in the RDP1 for grain As using the single‐experiment GWAS method.
**Table S3.** Summary of QTLs identified in the RDP1 for grain As using the multi‐experiment GWAS method.
**Table S4.** Comparison the QTLs for grain As in RDP1 identified in this study and when using a reduced number of SNPs.
**Table S5.** Identification of haplotypes in *OsPIP2;4*, *OsPIP2;1*, *OsPIP2;5*, and *OsPIP2;9*.
**Table S6.** Identification of haplotypes in *OsABCC1*.
**Table S7.** Summary of candidate genes identified this study.

**Figure S1** Distribution of the rice accessions by subpopulation.
**Figure S2.** Haplotype analysis of *OsPIP2;9*, *OsPIP2;5*, and *OsPIP2;1*.

## Data Availability

The phenotype data used to conduct the GWA mapping are available in the Supporting Information (Table S1).
