# Supplementary material for: Multi‐Experiment and Multi‐Locus Genome‐Wide Association Mapping for Grain Arsenic in Rice Population
Source: Plant Direct. 2025 May 4;9(5):e70064. doi: 10.1002/pld3.70064 (PMC12050220; doi:10.1002/pld3.70064)
Supplement: Supplementary file 2 — Figure S1 Distribution of the rice accessions by subpopulation. Figure S2. Haplotype analysis of OsPIP2;9, OsPIP2;5, and OsPIP2;1. [file PLD3-9-e70064-s001.docx]

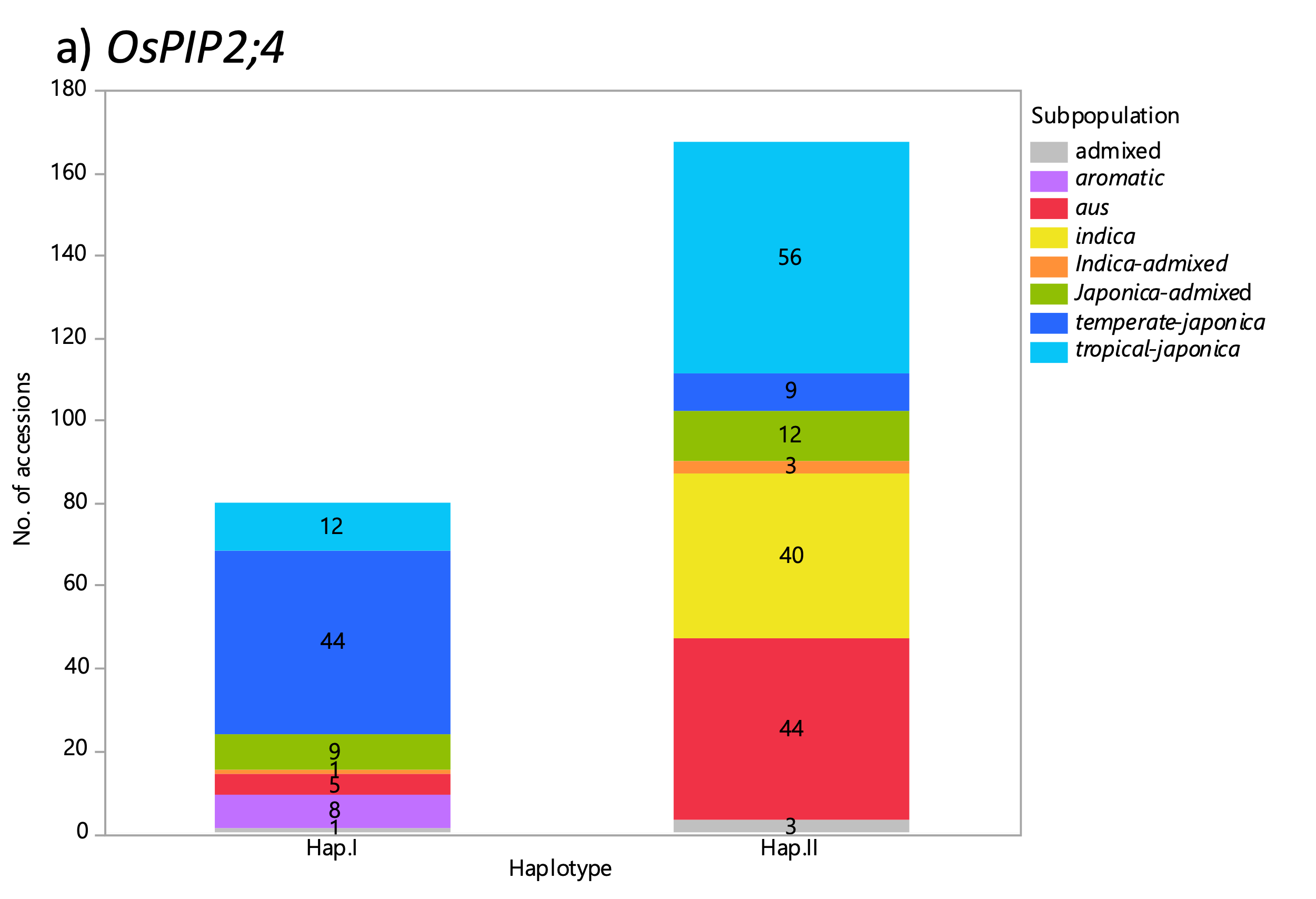


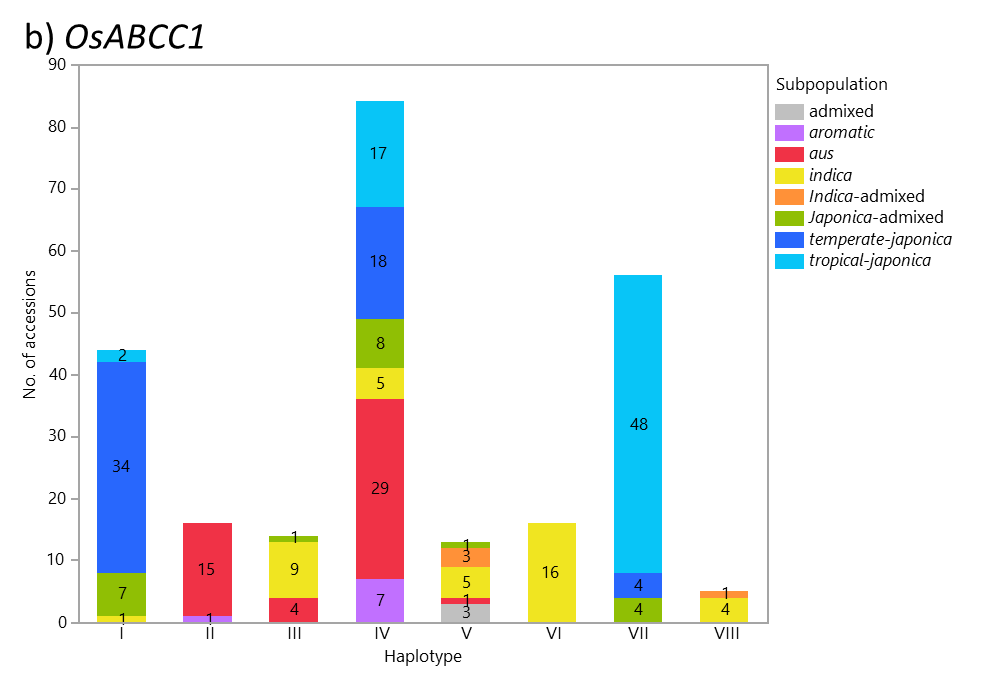


Supplementary Figure 1 Distribution of the rice accessions by subpopulation (McCouch et al., 2016) within the a) two haplotypes for *OsPIP2;4* listed in Supplementary Table 5 and b) eight haplotypes for *OsABCC1* listed in Supplementary Table 6


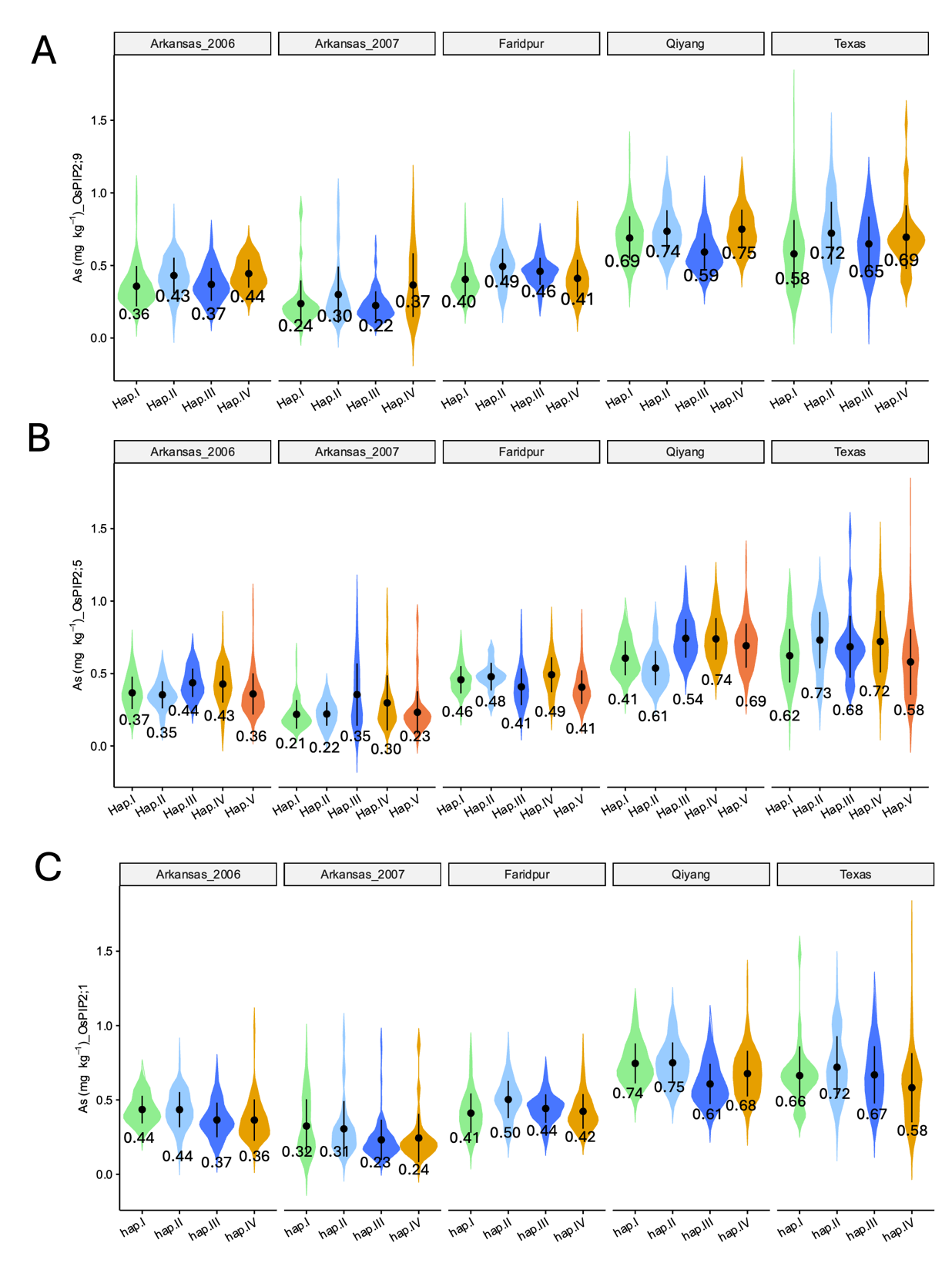


**Supplementary Figure 2. Haplotype Analysis of *OsPIP2;9, OsPIP2;5, and OsPIP2;1.***

(A) *OsPIP2;9* haplotype analysis: Cultivars were grouped into four haplotypes based on 11 SNPs—Haplotype I (n=76), II (n=48), III (n=79), and IV (n=37).

(B) *OsPIP2;5* haplotype analysis: Five haplotypes were identified based on 8 SNPs—Haplotype I (n=60), II (n=13), III (n=40), IV (n=50), and V (n=77).

(C) *OsPIP2;1* haplotype analysis: Four haplotypes were identified based on 4 SNPs—Haplotype I (n=35), II (n=50), III (n=84), and IV (n=75).

The data below each plot represents the mean arsenic level for each haplotype.
